# Patterns of Unilateral and Bilateral Projections From Layers 5 and 6 of the Auditory Cortex to the Inferior Colliculus in Mouse

**DOI:** 10.3389/fnsys.2021.674098

**Published:** 2021-10-21

**Authors:** Nathiya Vaithiyalingam Chandra Sekaran, Meena S. Deshpande, Baher A. Ibrahim, Gang Xiao, Yoshitaka Shinagawa, Daniel A. Llano

**Affiliations:** ^1^Department of Molecular and Integrative Physiology, University of Illinois at Urbana-Champaign, Champaign, IL, United States; ^2^Beckman Institute for Advanced Science and Technology, Urbana, IL, United States; ^3^Carle Illinois College of Medicine, Urbana, IL, United States

**Keywords:** auditory cortex, inferior colliculus, corticocollicular, corticotectal, auditory midbrain, Fluorogold, cholera toxin B, retrobeads

## Abstract

The auditory cortex sends massive projections to the inferior colliculus, but the organization of this pathway is not yet well understood. Previous work has shown that the corticocollicular projection emanates from both layers 5 and 6 of the auditory cortex and that neurons in these layers have different morphological and physiological properties. It is not yet known in the mouse if both layer 5 and layer 6 project bilaterally, nor is it known if the projection patterns differ based on projection location. Using targeted injections of Fluorogold into either the lateral cortex or dorsal cortex of the inferior colliculus, we quantified retrogradely labeled neurons in both the left and right lemniscal regions of the auditory cortex, as delineated using parvalbumin immunostaining. After dorsal cortex injections, we observed that approximately 18–20% of labeled cells were in layer 6 and that this proportion was similar bilaterally. After lateral cortex injections, only ipsilateral cells were observed in the auditory cortex, and they were found in both layer 5 and layer 6. The ratio of layer 5:layer 6 cells after lateral cortex injection was similar to that seen after dorsal cortex injection. Finally, injections of different tracers were made into the two inferior colliculi, and an average of 15–17% of cells in the auditory cortex were double-labeled, and these proportions were similar in layers 5 and 6. These data suggest that (1) only the dorsal cortex of the inferior colliculus receives bilateral projections from the auditory cortex, (2) both the dorsal and lateral cortex of the inferior colliculus receive similar layer 5 and layer 6 auditory cortical input, and (3) a subpopulation of individual neurons in both layers 5 and 6 branch to innervate both dorsal cortices of the inferior colliculus.

## Introduction

The auditory corticocollicular system consists of a large set of descending projections from the auditory cortex (AC) to the inferior colliculus (IC), which is the midbrain integration center (Suga, 2008; Malmierca and Ryugo, 2011; Bajo and King, 2012; Stebbings et al., 2014). The projection primarily targets the non-lemniscal nuclei of the IC: the dorsal cortex (DC) and lateral cortex (LC) (Saldaña et al., 1996; Winer et al., 1998; Torii et al., 2013). Stimulation and inactivation of AC inputs have been shown to have prominent effects upon the response properties of IC cells with respect to sound frequency and intensity, cues for spatial sound localization, and plastic changes in the IC (Jen et al., 1998; Yan and Suga, 1998; Popelář et al., 2003; Yan et al., 2005; Sun et al., 2007; Bajo et al., 2010; Asokan et al., 2018). The corticocollicular system is also heterogeneous and has been shown to emanate from two distinct layers of the AC: a large projection from layer 5 and a smaller (25% of the total in mice) projection from lower layer 6 (Schofield, 2009; Slater et al., 2019). Previous work has shown that neurons in these layers have different physiological properties, receive different cortical and thalamic inputs, and have different termination sizes in the IC (Slater et al., 2013, 2019; Yudintsev et al., 2019). Although the functional impact of layer 5 vs. layer 6 projections onto IC neurons is not yet known, in other corticofugal systems such as the corticothalamic projection, layer 5 and layer 6 neurons have different impacts on their synaptic targets, and likely have different roles in sensory processing (Ojima, 1994; Reichova and Sherman, 2004; Takayanagi and Ojima, 2006; Theyel et al., 2010; Williamson and Polley, 2019).

The corticocollicular system is one of multiple auditory corticofugal pathways that have cascading connectivity to ultimately influence auditory processing at the level of the auditory periphery (Xiao and Suga, 2002; Perrot et al., 2006; León et al., 2012; Terreros and Délano, 2015). Although these corticofugal systems appear to have many common organizational properties, one way in which they appear to differ is in the degree to which the projections are bilateral. Corticothalamic projections from AC to the medial geniculate body appear to be unilateral, whereas AC projections to the cochlear nucleus appear to be bilateral, but with an ipsilateral bias (Weedman and Ryugo, 1996; Jacomme et al., 2003; Schofield and Coomes, 2005). Studies have shown that corticocollicular neurons have a bilateral component in opossums, guinea pigs, and hedgehogs (Willard and Martin, 1984; Künzle, 1995; Saldaña et al., 1996; Coomes et al., 2005), with the majority of the projection being ipsilateral.

Despite the anatomical differences between layer 5 and layer 6 projections to the IC, many questions remain about the functional organization of this projection. For example, it is unknown in the mouse if both layer 5 and layer 6 project to both the DC and LC of the IC, and if so, if they project in equal proportions. In addition, it is unknown if both layer 5 and layer 6 project to both ICs in the mouse, and if so, if individual neurons branch to project to both sides. Therefore, in the current study, we injected a sensitive retrograde tracer, Fluorogold, unilaterally into either the DC or the LC of the mouse and determined the extent to which the projection was in the ipsi- vs. contralateral lemniscal AC fields. To determine if the bilaterality of the projection was due to individual neurons that branch to both ICs, or comprise separate ipsi- and contralateral projections, different tracers were placed in each IC, and examination was done for double-labeled cells in the AC. We found that while both LC and DC received layer 5 and layer 6 AC inputs in similar proportions, only the DC received bilateral inputs from the AC. In addition, we observed that after injections of different tracers to the two different ICs, double-labeled cells were observed in both layers 5 and 6, suggesting that a subset of cells from each layer branch to innervate each DC.

## Materials and Methods

### Animals

Experiments were performed in adult CBA/CaJ (Jackson Labs, 000654) or Swiss Webster (Envigo, Hsd:ND4) mice of both sexes ranging from ages 4 to 6 months. Swiss Webster mice were only used for the three injections involving red retrobeads. Thirteen mice were used in the quantitative analysis of this study, and their data are summarized in Tables 1, 2. Eight additional animals were excluded due to poor injection sites and were not included in the analysis. All procedures were approved by the Institutional Animal Care and Use Committee at the University of Illinois. Animals were housed in care facilities approved by the Association for Assessment and Accreditation of Laboratory Animal Care International. Every attempt was made to minimize the number of animals used and to reduce suffering at all stages of the study.

**TABLE 1:** All animals used for unilateral injections in this study, with numbers of cells counted per animal.

			**Ipsilateral**				**Contralateral**				
**Mouse**	**Tracer**	**Volume injected (nL)**	**Ipsi- Layer 5**	**Ipsi- Layer 6**	**Ipsi% Layer 5**	**Ipsi% Layer 6**	**Contra- Layer 5**	**Contra- Layer 6**	**Contra% Layer 5**	**Contra% Layer 6**	**Ratio Ipsi/Contra**
M1-DC	FG	40	1971	630	75.8	24.2	330	57	85.3	14.7	6.7
M2-DC	FG	40	1611	347	82.3	17.7	162	43	79.0	21.0	9.6
M3-DC	FG	40	731	230	76.1	23.9	126	21	85.7	14.3	6.5
M4-DC	FG	40	1193	195	86.0	14.1	265	64	80.6	19.5	4.2
M1-LC	FG	40	1217	376	76.4	23.6	0	0	0	0	–
M2-LC	FG	40	701	140	84.4	16.7	0	0	0	0	–
M3-LC	FG	40	1744	458	79.2	20.8	0	0	0	0	–
M4-LC	FG	40	1149	91	92.7	7.3	0	0	0	0	–

*Contra, contralateral; Ipsi, ipsilateral; FG, Fluorogold.*

**TABLE 2:** All animals used for bilateral injections in this study, with numbers of cells counted per animal.

**Mouse**	**Tracer**	**Volume injected (nL)**	**Layer 5 from Left**	**Layer 5 from Right**	**Layer 5 double label**	**Layer 5 % double labeled**	**Layer 6 from Left**	**Layer 6 from Right**	**Layer 6 double label**	**Layer 6 % double labeled**
M1-Bilat	FG Left	100	388	145	90	34.2%	93	66	29	22.3%
	CTB-555 Right	100								
M2-Bilat	FG Left	200	112	355	12	2.6%	15	17	2	6.7%
	CTB Right	300								
M4-Bilat	FG Left	200	415	285	127	22.2%	77	70	25	20.5%
	RB Right	300								
M5-Bilat	FG Left	200	523	636	111	10.6%	128	155	41	16.9%
	RB Right	300								
M6-Bilat	FG Left	200	353	574	76	8.9%	79	98	25	16.5%
	RB Right	300								

*Cells were pooled from two hemispheres. FG, Fluorogold; CTB, cholera toxin B; RB, red beads.*

### Tracer Injection

Sterile instruments and aseptic techniques were used for all surgical procedures. Mice were anesthetized intraperitoneally with a mixture of ketamine hydrochloride (100 mg/kg) and xylazine (3 mg/kg) and acepromazine (3 mg/kg). The mouse was placed into a Kopf Model 940 Small Animal Stereotaxic Instrument with digital readout. The head was shaved and disinfected with Povidone–iodide and 70% ethanol. An incision was made in the scalp and the surrounding skin was injected with lidocaine (2% Covetrus, United States) intradermally as local anesthetic and carprofen (3 mg/kg, Henry Schein Melville, NY, United States) was given subcutaneously for post-operative pain management. Moisture Eyes ophthalmic ointment was applied to each eye to protect the cornea from drying. A small craniotomy was made over the IC using a surgical drill, and a small glass micropipette (tip size approximately 10 microns) was filled with Fluorogold (Fluorochrome, LLC, Denver, CO, United States), 1% in phosphate-buffered saline (PBS) for unilateral injections, and 0.1 acetate buffer (pH 3.3) for the bilateral injections. The left side was chosen for consistency, given left–right differences that have been described in the mouse auditory system (Oviedo et al., 2010; Levy et al., 2019). For bilateral injections, the right IC was injected with either Cholera Toxin B conjugated to Alexa-Fluor 555 (CTB-555, Invitrogen Cat# C34776) at 1% in PBS, Lumafluor red retrobeads (RB, diluted 1:1.5 in PBS), or unconjugated CTB (Listlabs #104, 2 mg/mL in PBS). In all cases, tracers were pressure injected into the IC using a WPI Nanoliter 2010 injector and Micro4 pump controller at 10–20 nl per min. Volumes of injectates varied and are shown in Tables 1, 2. After awakening from surgery, animals were returned to their home cages and to their vivarium until euthanized.

### Tissue Processing

Following a 7-day survival period, animals were anesthetized with overdose of ketamine and xylazine (200 mg/kg, 6 mg/kg) and perfused transcardially with 4% paraformaldehyde (PFA) in PBS at pH 7.4. Brains were removed and post fixed overnight in the PFA solution. After being cryoprotected in an ascending series of sucrose solutions, each brain was embedded and cut into 40–50-μm-thick coronal sections on a cryostat that were collected serially in two sets.

### Immunostaining

Parvalbumin (PV) immunostaining was done to delineate the borders of the lemniscal regions of the AC [primary AC and anterior auditory field (Molinari et al., 1995; Kosaki et al., 1997; Cruikshank et al., 2001; Llano and Sherman, 2008)]. Sections were microwaved for 15 s and then incubated for 30 min in a solution of 0.3% Triton X-100 in PBS to enhance membrane permeability. The sections were then transferred to a blocking solution consisting of 0.3% Triton X-100 and 3% goat serum in PBS and incubated for 30 min. The primary antibody solution consisted of 1:500 monoclonal anti-PV raised in mouse (P 3088, Sigma Aldrich) in the blocking solution. Sections were incubated in this solution overnight and rinsed in three changes of the Triton X-100 in PBS solution the following day. The sections were then transferred to a secondary antibody solution and incubated at room temperature for 2 h. This solution consisted of 1:100 Alexa Fluor 568-conjugated goat anti-mouse secondary antibody (catalog #A-11004, Invitrogen). Following a final series of washes in PBS, the sections were mounted on gelatin-coated slides and coverslipped with an anti-fade solution (Vectashield; Vector Laboratories). For immunostaining of CTB, after blocking with 3% donkey serum in 0.3% Triton X-100, sections were incubated with 1:10,000 anti-CTB antibody in blocking solution (#703 Listlabs) overnight at 4°C. After washing, sections were incubated with 1:200 solution of Donkey anti-Goat IgG (H + L) Cross-Adsorbed Secondary Antibody conjugated to Alexa Fluor 555 (Invitrogen # A-21432) diluted in blocking buffer with a 1:200 dilution.

### Imaging and Analysis

Sections were imaged with a Leica SP8 laser scanning confocal microscope and LAS X control software or with an Olympus IX71 inverted epifluorescence microscope. For confocal images, each IC tissue section containing retrograde label, 40 × mosaic Z-stacks were taken throughout the entire depth and *x*–*y* plane of the IC. The stacks were collapsed into 2D maximum intensity projections and tiled into a single image using LAS X software. ImageJ software was used to adjust the color balance and to draw masks around the edge of the tissue to remove the embedding medium.

### Data Quantification and Statistics

PV immunostaining was used to mark the borders of the lemniscal portions of the AC. For counting Fluorogold-labeled cells in the AC, neurons from six non-consecutive sections were selected. These sections were distributed across the anterior–posterior dimension of the AC. The labeled cells in AC within the PV-enriched zone were counted using ImageJ software. For counting double-labeled cells, to ensure that areas of maximum overlap were examined, only sections where at least 20 cells per tracer type were present were analyzed. Given the small numbers of animals (*n* = 4 in each injection location for unilateral injections, *n* = 5 for bilateral tracer injections), normality was not assumed and non-parametric statistics were used throughout with a threshold for significance of *p* < 0.05. Data are presented as median ± standard deviation.

## Results

**FIGURE 1:**
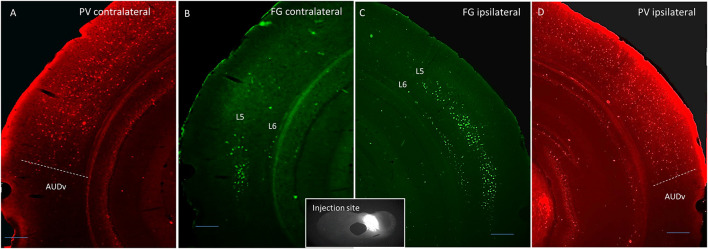
Coronal images showing an example DC injection site (inset), and the corresponding contralateral Fluorogold label **(B)**, ipsilateral Fluorogold label **(C)**, and PV immunostaining pattern **(A,D)**. Scale bar = 250 μm. L5 = layer 5, L6 = layer 6, AUDv, ventral auditory region.

Thirteen adult (4–6 months old) male and female mice were used in this study. Four were injected with Fluorogold into the left LC, four were injected into the left DC, and five were injected into both ICs. For both DC and LC injection sites, there tended to be some spillover into the central nucleus of the IC (CNIC). See Tables 1, 2 for a listing of all mice used for quantitative analysis in this study. For the mice injected into either DC or LC, PV immunostaining was done to delineate the lemniscal regions of the AC. An example of a DC injection site as well as images from both ACs demonstrating layer 5 and layer 6 retrograde label, and the corresponding PV images are shown in Figure 1.

### Distributions of Ipsi- vs. Contralateral Cells in Auditory Cortex After Dorsal Cortex or Lateral Cortex Tracer Injection

The total number of cells in the lemniscal regions of the ipsilateral and contralateral AC were counted and compared. In all cases of DC injections (*n* = 4), there were significantly greater numbers of retrogradely labeled cells in the ipsilateral cortex (*p* = 0.02, Wilcoxon signed-rank test), with a median ipsilateral:contralateral ratio = 6.63 ± 2.18, indicating that approximately 85% of all labeled cells were ipsilateral. After injection into the LC (*n* = 4), 100% of all labeled cells in all animals were found in the ipsilateral AC.

### Distribution of Layer 5 vs. Layer 6 Corticocollicular Cells in Ipsi- vs. Contralateral Dorsal Cortex Injection

**FIGURE 2:**
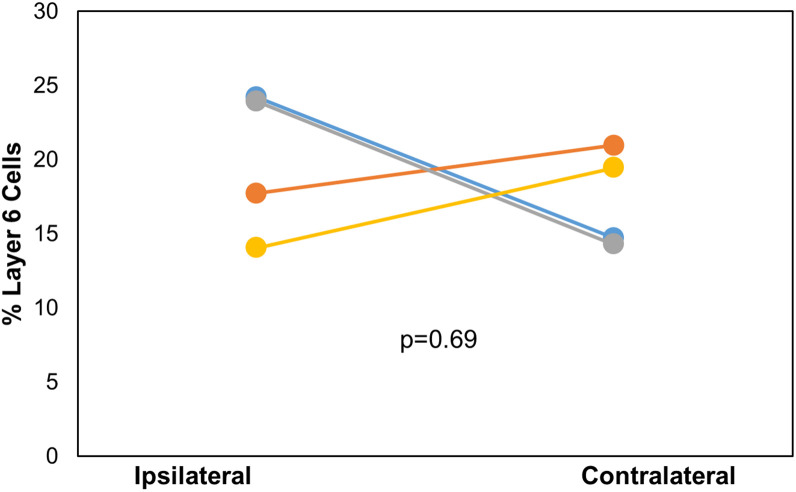
Comparison of the layer 6 percent of total cells located ipsilaterally and contralaterally of the lemniscal AC between each mouse (*n* = 4) with injection site in DC. *p*-value calculated using Wilcoxon Signed Rank Test. See Table 1 for actual values. *n* = 4 mice. Each color corresponds to a different mouse.

After DC injection, the proportions of layer 5 vs. layer 6 corticocollicular cells in the lemniscal AC were compared using a within-animal comparison. Although the total number of cells was significantly greater on the ipsilateral side, there was no difference in the proportion of layer 6 cells on the two sides (ipsilateral: 20.83 ± 4.96% vs. contralateral: 17.09 ± 3.36%, *p* = 0.69, Wilcoxon signed-rank test, Figure 2). Although LC injections did not produce any contralateral label, the retrogradely labeled cells in the ipsilateral lemniscal AC contained substantial layer 6 label. We compared the proportion of ipsilateral layer 6 label after DC compared to LC injections and found no significant difference between the two (DC 20.83 ± 4.96%, LC 18.72 ± 7.11%, *p* = 0.49, Mann–Whitney).

### Double-Retrograde Injection Into Left and Right Lateral Cortex

**FIGURE 3:**
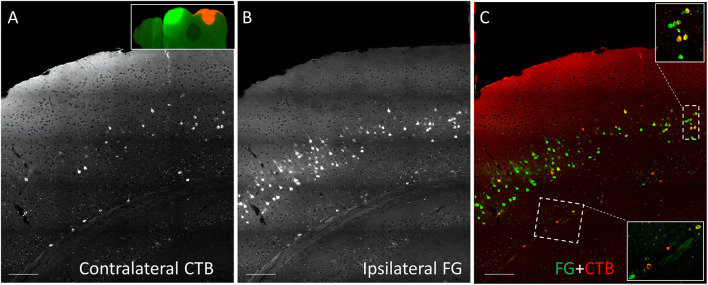
Coronal sections showing double injection site of Fluorogold (FG) on the left and CTB-555 on the right IC. **(A)** Grayscale image showing contralateral CTB-555-labeled cells. **(B)** Grayscale image showing ipsilateral FG-labeled cells. **(C)** Overlay between panel **(A)** and panel **(B)**, showing single-labeled (red or green) and double-labeled (yellow) cells in each layer in insets. Scale bar = 100 μm.

**FIGURE 4:**
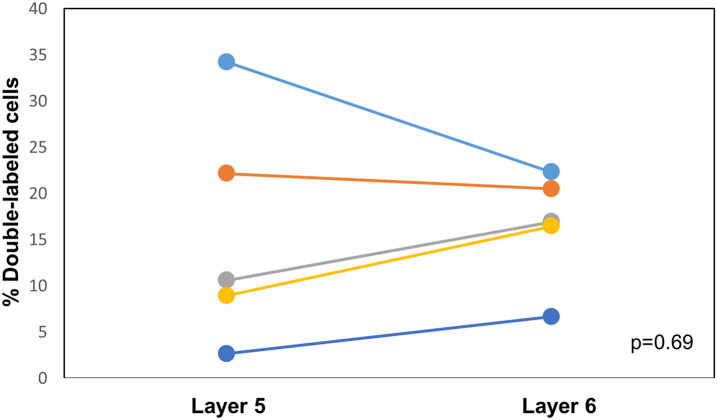
Comparison of the proportion of double-labeled cells found in each layer after injection of different tracers into the left and right IC. See Table 2 for actual values. *n* = 5 mice. *p*-value calculated using Wilcoxon Signed Rank Test. Each color corresponds to a different mouse.

CTB-555, unconjugated CTB, or RB were injected into the right IC and Fluorogold was injected into the left IC, and images containing cells stained with each tracer were overlaid. Similar to previous findings (Coomes et al., 2005), we found three cell labeling types in each layer: CTB- or RB-only labeled cells, Fluorogold-only labeled cells, and cells labeling with both a red tracer and Fluorogold. All three cell types were found in both layer 5 and layer 6 (Figure 3), suggesting the presence of branching cells in each layer. The numbers of single- and double-labeled cells in each layer were counted and summarized in Table 2. The proportions of double-labeled cells ranged from 2.6 to 34.2% per layer per animal. The mean proportion of double-labeled cells was similar in layer 5 compared to layer 6 (15.7 ± 12.5 [SD]% in layer 5 vs. 16.6 ± 6.1 [SD]% in layer 6, *n* = 5 mice, *p* = 0.686, Wilcoxon Signed Rank, see Figure 4).

## Discussion

**FIGURE 5:**
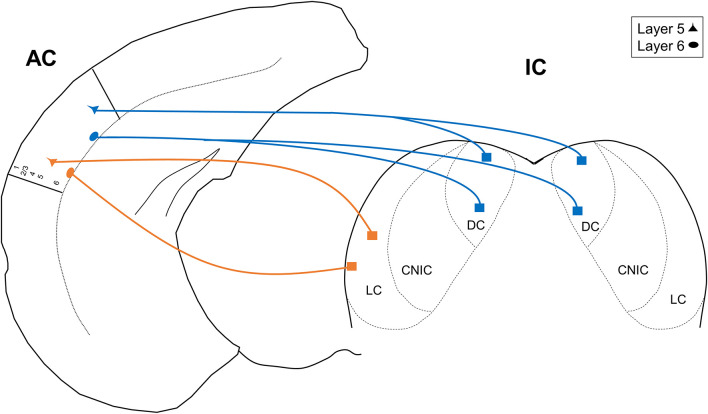
Model depicting the unilateral and projections from layers 5 and 6 of the AC. CNIC, central nucleus of the IC.

In this report, we used single- and double-retrograde tracing methods in the mouse to investigate patterns of unilateral vs. bilateral input from AC layers 5 and 6 to the IC. We report three main findings: (1) only the DC receives bilateral projections from the AC, (2) both the DC and LC receive similar proportions of layer 5 and layer 6 auditory cortical input, and (3) a subset of individual neurons in both layers 5 and 6 branch to innervate the DC bilaterally. These findings are summarized in the model shown in Figure 5. Below, we discuss the implications of these findings.

### Technical Considerations

Cortical areas in this study were defined using PV immunostaining, which has been established to distinguish lemniscal auditory areas (primary AC and anterior auditory field) from non-lemniscal areas (Molinari et al., 1995; Kosaki et al., 1997; Cruikshank et al., 2001). Thus, we have not attempted to differentiate primary AC vs. anterior auditory field proportions of either layer 5 or layer 6, although we consistently see similar proportions of each cell type throughout the anterior–posterior extent of the PV-enriched zone. Future work using *in vivo* mapping of auditory fields prior to the injection of tracers may be helpful to determine if differences exist in the layer 5 vs. 6 projections from primary AC or anterior auditory field to the IC, or in the non-lemniscal regions of the auditory cortex.

The proportion of cells found to be double-labeled after injection of different tracers into different sides was found to be relatively low (range = 2.6–34.2%). Previous work has shown that this approach is susceptible to significant undercounting (Doucet et al., 2003; Coomes et al., 2005; Schofield et al., 2007). For example, when co-injecting mixed tracers of different chemical entities, one of which being synthetic beads (similar to the current study) into the same location of the IC, a range of 4–70.1% of AC cells were double-labeled (Schofield et al., 2007). The undercounting occurs because the two injections may not be matched in terms of their projection fields and because of differential efficiency of the two labels. We attempted to make our sites large enough to encompass major portions of the DC bilaterally, but without being so large as to risk entering neighboring structures, such as superior colliculus. Thus, we assume that the percentage of cells that branch to innervate both colliculi is higher than the proportion of double-labeled cells reported here.

### Implications

The results of this study suggest that both the DC and LC receive similar proportions of layer 5 and layer 6 input, but that only DC receives input from the contralateral AC. These results differ somewhat from those of Schofield (2009), who did not observe contralateral corticocollicular projections from layer 6 in guinea pig. The differences may be related to the different species or tracer used. Although layer 6 projections to the IC have been identified in multiple species including mice, rats, gerbils, ferrets, and hedgehog tenrec (Games and Winer, 1988; Künzle, 1995; Doucet et al., 2003; Bajo and Moore, 2005; Bajo et al., 2007), the relative proportions of those projections may differ. In the case of guinea pigs, approximately 10.2% of the total cell population was determined to come from layer 6 using a variety of tracers excluding Fluorogold. However, the current study and a previous study have established that this number is roughly 20–25% in mice using Fluorogold, which is a very sensitive retrograde tracer (Schofield, 2008). This difference in the tracers, and the approximately sixfold greater ipsilateral- vs. contralateral-projecting corticocollicular cells than contralateral-projecting cells, coupled with a smaller proportion of layer 6 cells in guinea pigs, suggest that the observation of the lack of layer 6 contralateral-projecting cells in guinea pig was due to a threshold effect.

We also observed that a small proportion of cells branched to innervate both ICs (15.7% in layer 5 and 16.6% in layer 6). These values are higher than those seen in the guinea pig layer 5 [range = 2.5–11.9% (Coomes et al., 2005)]. However, given the uncertainties regarding the precise values of double-labeled cells as outlined by several authors previously (Doucet et al., 2003; Coomes et al., 2005; Schofield et al., 2007) as well as the species and tracer differences in this study, it is not clear that the differences between our study and the Coomes et al. (2005) study represent real biological differences in branching patterns. However, both studies do indicate that bilateral coordination of IC modulation is an important feature in a subset of the corticocollicular projection, and the current report extends this finding to layer 6.

The implications of having bilateral projections from AC to portions of the IC are not yet known. The IC receives bilateral projections from the auditory brainstem (Coleman and Clerici, 1987; Cant and Benson, 2006, 2008) and receives inputs from the superior olive (Kelly et al., 1998; Loftus et al., 2004), which itself gets bilateral input. Therefore, it is unlikely that a bilateral descending projection is required to produce sensitivity to sounds from both ears. The layer 5 corticofugal system has been hypothesized to serve as a system that drives rapid escape behaviors (Xiong et al., 2015; Li et al., 2021). In addition, the layer 5 corticofugal projections in other sensory systems appear to be widely branching to multiple sensory and motor regions (Deschênes et al., 1994; Bourassa and Deschenes, 1995; Bourassa et al., 1995; Kita and Kita, 2012; Guo et al., 2017; Prasad et al., 2020). Therefore, it is not surprising to find a bilateral projection system to the inferior colliculi, which also project to motor structures to mediate escape responses (Kawamura, 1975; Aitkin and Boyd, 1978; Edwards et al., 1979; Cadusseau and Roger, 1985; Appell and Behan, 1990; Huffman and Henson, 1990; Lesicko and Llano, 2020). The presence of a bilateral layer 6 system, which we have previously speculated to serve a modulatory role (Yudintsev et al., 2019; Asilador and Llano, 2020), was less expected, but may suggest that the dual functions of layer 5 and layer 6 are necessary for the potential escape function of the layer 5 corticofugal projections. The absence, then, of a bilateral projection to the LC may suggest that this region is less likely to be involved in rapid motor escape behaviors than DC. Future work comparing the corticofugal response properties of cortical-recipient cells in LC or DC will help to clarify their separate roles in acoustic behavior.

### Summary and Conclusion

In this study, we observed that lemniscal regions of the AC send bilateral projections from layers 5 and 6 to the DC of both inferior colliculi, with the majority being ipsilateral. We also observed that the LC receives only an ipsilateral projection from the AC and that this projection is derived from both layers 5 and 6. The proportion of layer 6 cells projecting to the IC is approximately 18–20% and does not differ based on IC target. Finally, we observed that the bilateral projection to the DC comprises, at least in part, individual neurons in both layers 5 and 6 that branch to innervate both DCs. Understanding the implications of these findings requires further investigation but may relate to the suspected roles of corticofugal projections in rapid acoustic escape behaviors.

## Data Availability Statement

The original contributions presented in the study are included in the article/supplementary material, further inquiries can be directed to the corresponding author/s.

## Ethics Statement

The animal study was reviewed and approved by Illinois Institutional Animal Care and Use Committee (IACUC).

## Author Contributions

NV, MD, and DL analyzed the data and co-wrote the manuscript. BI performed stereotactic injections. GX and YS processed the tissue sections. All authors contributed to the article and approved the submitted version.

## Conflict of Interest

The authors declare that the research was conducted in the absence of any commercial or financial relationships that could be construed as a potential conflict of interest.

## Publisher’s Note

All claims expressed in this article are solely those of the authors and do not necessarily represent those of their affiliated organizations, or those of the publisher, the editors and the reviewers. Any product that may be evaluated in this article, or claim that may be made by its manufacturer, is not guaranteed or endorsed by the publisher.
